# Analysis of changes on adiponectin levels and abdominal obesity after smoking cessation

**DOI:** 10.1371/journal.pone.0201244

**Published:** 2018-08-07

**Authors:** Maki Komiyama, Hiromichi Wada, Hajime Yamakage, Noriko Satoh-Asahara, Yoichi Sunagawa, Tatsuya Morimoto, Yuka Ozaki, Akira Shimatsu, Yuko Takahashi, Koji Hasegawa

**Affiliations:** 1 Clinical Research Institute, National Hospital Organization Kyoto Medical Center, Kyoto, Japan; 2 Division of Molecular Medicine, School of Pharmaceutical Sciences, University of Shizuoka, Shizuoka, Japan; International University of Health and Welfare, School of Medicine, JAPAN

## Abstract

**Purpose:**

The blood levels of Adiponectin, anti-inflammatory and anti-arteriosclerotic adipocytokine, decrease due to smoking and obesity. Cigarette smokers are generally known to gain weight after smoking cessation (SC). Nevertheless, precise changes in serum adiponectin levels after SC and specific effects of abdominal obesity on those changes remain unknown. The objective of this study was to elucidate the changes in serum adiponectin levels after SC and the effects of abdominal obesity on those changes.

**Methods:**

In 86 patients (56 males and 30 females) who had successfully quit smoking, serum adiponectin levels were measured using an enzyme-linked immunosorbent assay at baseline and 1 year after beginning SC.

**Results:**

Body mass index and waist circumference (WC) were significantly increased 1 year after beginning SC. Adiponectin levels, however, did not change after SC. Using the median ΔWC (+2.8%) as the cutoff point, patients were then divided into two groups. The percent change in adiponectin levels from baseline to 1 year was significantly greater in the ΔWC < median group (−1.1%) than in the ΔWC ≥ median group (+8.1%) (*p* = 0.011).

**Conclusions:**

Despite weight gain and increased abdominal obesity, serum adiponectin levels did not decrease after SC. Therefore, the beneficial effect of SC may eliminate the adverse effects of subsequent weight gain. Conversely, patients with less abdominal obesity had increased adiponectin levels 1 year after SC. Therefore, in such patients, the beneficial effect of SC on adiponectin levels is apparent.

## Introduction

Although cardiovascular disease risk decreases within two years of smoking cessation, ≥10 years may be needed for risk levels to match those in nonsmokers [1]. Moreover, smokers, who typically have higher levels of high-sensitivity CRP, may need 20 years to significantly decrease high-sensitivity CRP levels to match those in nonsmokers [2]. It remains unclear why such a prolonged period of time is needed for cardiovascular risk to decrease to levels found in nonsmokers after smoking cessation. One reason may be increased oxidative stress due to weight gain after smoking cessation. Weight gain typically occurs for about three years after smoking cessation and has been reported to worsen glucose tolerance [3–5]. In addition, worsening glucose tolerance has been known to be proportional to the extent of weight gain [4, 6, 7]. Weight gain after smoking cessation should be limited to 5 kg to significantly reduce the risk of cardiovascular events in smokers with diabetes mellitus [8]. Therefore, the advantages of smoking cessation and the disadvantages of gaining weight intricately coexist for several years after smoking cessation. To maximize the reduction in risk due to smoking cessation, comprehensive management (including management of weight gain after smoking cessation) is required.

Adipocytokines, which are endocrine factors produced and secreted by adipocytes, have been associated with the pathophysiology of obesity. One type of adipocytokine, adiponectin, is considered to be a hormone with anti-inflammatory [9, 10], anti-arteriosclerotic [11–13], and metabolism-improving effects [14]. Adiponectin also functions to directly protect against cardiovascular disease [15]. Adiponectin levels in the blood decrease due to obesity, particularly due to increased visceral fat [16]. Studies have shown that a decrease in adiponectin causes decreased anti-inflammatory and increased pro-inflammatory adipocytokine expression, leading to the progression of obesity-related diseases, such as cardiovascular disease. Studies have also reported that patients with coronary artery disease have lower serum adiponectin levels than healthy individuals [11], while individuals with lower serum adiponectin levels have about 2.1 times the risk of developing coronary artery disease compared to individuals with normal levels [17]. These findings suggest that adiponectin could be a useful predictive biomarker for coronary artery disease.

In addition, adiponectin has been closely related to smoking. Studies have reported that smokers have about 30% lower serum adiponectin levels than nonsmokers [18]. Another study reported that the adiponectin level in the blood of ex-smokers is higher than that in smokers, but lower than that in individuals who never smoked. In addition, another study reported that adiponectin levels in blood remained elevated for three months after an individual quit smoking [19]. A further study reported that weight typically increases for about three years after quitting smoking [20], and another study indicated that weight gain is associated with a decrease in adiponectin level. Even with the abundance of published research, precise changes in serum adiponectin levels after smoking cessation and detailed aspects of the relationship between those changes and weight gain or abdominal obesity after smoking cessation remain unknown.

### Objective

The aim of the current study was to elucidate the changes in serum adiponectin levels after smoking cessation and the effects of abdominal obesity on those changes.

## Materials and methods

### Participants

This is a prospective study which was conducted at the National Hospital Organization, Kyoto Medical Center during the period of April 2009 to January 2014. Patients who consulted the smoking cessation clinic to receive treatment and successfully quit smoking for 1 year were enrolled in this study. Individuals, other than those who met the exclusion criteria, were not arbitrarily excluded. The exclusion criteria were as follows: concomitant acute coronary syndrome; infection or pyrexia; recent (<3 months) myocardial infarction or stroke; renal transplantation or serum creatinine ≥ 3 mg/dL; hepatic failure, defined as chronic hepatic disease (i.e., cirrhosis) or biochemical evidence of significant hepatic derangement (e.g., bilirubin >3 × upper limit of normal, in association with aspartate aminotransferase/alanine aminotransferase/alkaline phosphatase >3 × upper limit normal); and active inflammatory diseases. Various parameters were evaluated in these patients at the time of initial consultation and after smoking cessation (at 1 year after the initial consultation). Informed written consent was obtained from all participants. They were not coerced into taking part in this study. The study data was anonymized with no personal identifiers. The Ethical Review Board, National Hospital Organization, Kyoto Medical Centre approved the study protocol. All procedures performed were in accordance with the ethical standards of the institutional and national research committee and with the 1964 Helsinki declaration and its later amendments or comparable ethical standards.

### Smoking cessation clinic and data collection

The details are described elsewhere [21]. Briefly, anti-smoking treatment was conducted according to the Standard Procedures for Anti-Smoking Treatment (originally issued in March 2006 by the Japanese Circulation Society, Japan Lung Cancer Society, and Japanese Cancer Association) [22]. Blood tests were conducted three times at their first consultation as a screening and at 1 year after their first visit to assess the change of the biochemical and hematological profile of patients. Serum levels of leptin and adiponectin were measured employing specific sandwich enzyme-linked immunosorbent assays (ELISA) (Ikagaku Co., Ltd., Kyoto, Japan). Serum levels of high-molecular-weight (HMW) adiponectin were measured employing specific Quantikine ELISA Human HMW Adiponectin/Acrp30 Immunoassay (R&D systems).

### Statistical analysis

All statistical analyses were performed by a professional statistician using the Statistical Package for Social Sciences (SPSS) Statistics 17.0 statistical software package (SPSS Inc., Chicago, IL, USA). The normality was assessed using the Shapiro-Wilk test. Clinical data before and after successful smoking cessation were compared using the paired t-tests for parametric data or the Wilcoxon signed rank test for non-parametric data. In addition, changes in data from before to after smoking cessation were compared by the unpaired t-tests for parametric data or the Mann-Whitney U test for non-parametric data between patients with smaller versus larger waist circumference (WC) changes. In addition, changes in data from before to after smoking cessation were compared by two-way analysis of variance between patients with a WC increase smaller than the median and those with a WC increase greater than the median.

## Results

### Participants

Among the 783 patients who visited the smoking cessation clinic to receive treatment and consented to study participation, 408 successfully quit smoking 3 months after their first visit. Among the 101 patients who revisited the smoking cessation clinic 1 year after their first visit, 3 experienced relapse. Among the 98 patients who successfully continued smoking cessation 1 year after their first visit, 12 patients were excluded because of the lack of blood sampling data. Ultimately, we were able to analyze the findings of 86 patients [56 males and 30 females aged between 34 and 80 years (mean 61 ± 13 years)]. WC measurements were missing for seven participants. Various parameters were evaluated in these patients upon initial consultation and 1 year thereafter. The participants had an average FTND score of 6.5 ± 2.3, a daily number of cigarettes smoked of 23.2 ± 10.7, and a Brinkman index of 885 ± 462. Expired CO concentrations significantly decreased from the initial visit to 1 year after smoking cessation (from 14.1 to 1.7, *p* < 0.0001).

### Changes in adiponectin and leptin levels and metabolic parameters

BMI and WC significantly increased from baseline to 1 year after beginning smoking cessation as shown in Table 1. Leptin levels significantly increased from 3.2 μg/mL to 4.8 μg/mL (*p* < 0.001) in proportion to weight gain. Total adiponectin levels, however, did not change after smoking cessation (from 8.1 to 8.3 μg/mL, *p* = 0.300). HWM adiponectin levels did not change after smoking cessation neither (from 1.8 to 1.9 μg/mL, *p* = 0.115). The proportion of HMW Adiponectin relative to total Adiponectin (HMW/total Adiponectin) did not change after smoking cessation (*p* = 0.126). Correcting for WC at each time of measurement revealed a significant increase in adiponectin levels in the blood after quitting smoking (from 7.8 to 8.4 μg/mL, *p* = 0.047).

**Table 1:** Patient data before and 1 year after successful smoking cessation (n = 86).

	Before	After	*p-*value	
**BMI (kg/m**^**2**^**)**	**23.1 [21.5, 25.3]**	**24.4 [22.2, 26.7]**	**<0.001**	**a**
**WC (cm)**	**86.6 ± 9.9**	**89.4 ± 9.5**	**<0.001**	**b**
SBP (mmHg)	131 ± 16	127 ± 18	0.070	b
DBP (mmHg)	76 ± 11	75 ± 11	0.801	b
HbA1c (%)	5.8 [5.6, 6.1]	5.8 [5.6, 6.2]	0.364	a
LDL-C (mg/dL)	116 ± 30	115 ± 31	0.818	b
HDL-C (mg/dL)	53 [45, 63]	57 [45, 68]	0.073	a
TG (mg/dL)	137 [99, 201]	150 [119, 213]	0.088	a
**Leptin (**μ**g/mL)**	**3.2 [1.5, 6.9]**	**4.8 [2.2, 9.1]**	**<0.001**	**a**
Total adiponectin (μg/mL)	8.1 ± 3.2	8.3 ± 3.7	0.300	b
HMW adiponectin (μg/mL)	1.8 [1.0, 2.7]	1.9 [1.0, 3.1]	0.115	a
HMW/total adiponectin (%)	21 [15, 27]	22 [16, 28]	0.126	a
hsCRP (mg/dL)	0.8 [0.3, 2.5]	0.7 [0.3, 1.8]	0.722	a

Data are presented as mean ± standard deviation or median [interquartile range].

*p-*value: a, Wilcoxon signed rank test; b, paired t-test

BMI, body mass index; WC, waist circumference; SBP, systolic blood pressure; DBP, diastolic blood pressure; HbA1c, hemoglobin A1c; LDL-C, low-density lipoprotein cholesterol; HDL-C, high-density lipoprotein cholesterol; TG, triglycerides; HMW, high molecular weight; HMW/total adiponectin, the ratio of the HMW adiponectin levels to the total adiponectin levels; hsCRP, high sensitivity C-reactive protein

Using the median ΔWC (+2.8%) as the cutoff point, patients were divided into two groups. The mean ΔWC change rate was −1.1% in the ΔWC < median group (n = 39, 28 males and 11 females) and +8.1% in the ΔWC ≥ median group (n = 40, 23 males and 17 females). Regarding prescription medicines, 18 and 21 individuals used a nicotine patch and varenicline in the ΔWC < median group, whereas 19 and 21 individuals used a nicotine patch and varenicline in the ΔWC ≥ median group, respectively. Items assessed during the initial visit were compared between both groups, the results of which are shown in Table 2. Patients in the ΔWC ≥ median group were younger (*p* = 0.012), had smoked for less time (in years) (*p* = 0.036), and tended to have higher FTND scores (*p* = 0.067) than those in the ΔWC < median group.

**Table 2:** Patient data before smoking cessation: Comparison between patients with smaller versus larger waist circumference changes.

	ΔWC (%) < median^†^	ΔWC (%) ≥ median	*p-*value	
Male	71.8% (28/39)	57.5% (23/40)	0.241	a
Age (years)	65 ± 11	57 ± 13	0.012	b
Daily cigarette consumption (n)	20 [15, 25]	20 [20, 30]	0.144	c
Smoking years	42 ± 11	36 ± 12	0.036	b
FTND score	6.0 ± 2.5	7.0 ± 2.3	0.067	b

Data are presented as the mean ± standard deviation or median [interquartile range]

^†^median ΔWC = 2.8%

*p-*value: a, Fisher’s exact test; b, unpaired t-test; c, Mann−Whitney U test

FTND, Fagerström Test for Nicotine Dependence

As shown in Table 3, the ΔWC < median group (−1.1%) experienced a significant decrease in SBP and increase in HDL-C after 1 year. In the ΔWC ≥ median group (+8.1%), metabolic parameters worsened after smoking cessation. Regarding adipocytokines, leptin levels increased in the ΔWC ≥ median group but not in the ΔWC < median group. Moreover, total adiponectin and HMW adiponectin levels increased significantly in the ΔWC < median group (*p* = 0.009 and *p* < 0.001, respectively) but did not increase in the ΔWC ≥ median group after smoking cessation. In addition, HMW/total adiponectin also increased significantly in the ΔWC < median group (*p* < 0.001) but did not increase in the ΔWC ≥ median group after smoking cessation.

**Table 3:** Patient data before and 1 year after successful smoking cessation in the lesser (A) and greater (B) abdominal obesity groups.

**A:** Δ**WC (%) < median**^†^**, n = 39**				
	Before	After	*p* value	
BMI (kg/m^2^)	24.0 [22.1, 26.0]	24.3 [22.1, 26.1]	0.338	a
WC (cm)	89.9 ± 9.3	89.0 ± 10.1	0.093	b
**SBP (mmHg)**	**134 ± 14**	**127 ± 16**	**0.023**	**b**
DBP (mmHg)	78 ± 8	75 ± 10	0.064	b
HbA1c (%)	5.9 [5.6, 6.3]	5.8 [5.6, 6.3]	0.103	a
LDL-C (mg/dL)	119 ± 31	110 ± 27	0.052	b
**HDL-C (mg/dL)**	**50 [42, 57]**	**54 [45, 67]**	**0.011**	**a**
TG (mg/dL)	148 [115, 231]	139 [116, 234]	0.676	a
Leptin (μg/mL)	3.4 [1.5, 7.1]	4.6 [1.7, 7.5]	0.061	a
**Total adiponectin (**μ**g/mL)**	**7.5 ± 3.1**	**8.3 ± 3.7**	**0.009**	**b**
**HMW adiponectin (**μ**g/mL)**	**1.6 [1.0, 2.7]**	**2.0 [1.0, 3.7]**	**<0.001**	**a**
**HMW/total adiponectin (%)**	**18.7 [15.2, 28.1]**	**22.9 [15.0, 31.7]**	**<0.001**	**a**
hsCRP (mg/dL)	0.9 [0.4, 2.7]	0.9 [0.4, 1.8]	0.364	a
**B:** Δ**WC (%) ≥ median**^†^**, n = 40**				
	Before	After	*p* value	
**BMI (kg/m**^**2**^**)**	**23.1 [20.7, 24.5]**	**25.3 [22.3, 27.0]**	**<0.001**	**a**
**WC (cm)**	**83.4 ± 9.5**	**89.9 ± 9.1**	**<0.001**	**b**
SBP (mmHg)	127 ± 17	129 ± 19	0.491	b
DBP (mmHg)	73 ± 12	76 ± 13	0.134	b
**HbA1c (%)**	**5.8 [5.5, 6.0]**	**5.8 [5.6, 6.2]**	**0.006**	**a**
LDL-C (mg/dL)	112 ± 30	119 ± 33	0.188	b
HDL-C (mg/dL)	56 [47, 68]	60 [47, 70]	0.864	a
**TG (mg/dL)**	**112 [80, 176]**	**153 [119, 185]**	**0.011**	**a**
**Leptin (**μ**g/mL)**	**3.2 [1.4, 6.8]**	**5.5 [2.7, 13.9]**	**<0.001**	**a**
Total adiponectin (μg/mL)	8.4 ± 3.3	8.3 ± 3.7	0.536	b
HMW adiponectin (μg/mL)	1.9 [1.1, 2.9]	1.8 [1.1, 2.3]	0.586	a
HMW/total adiponectin (%)	21.2 [16.3, 25.1]	21.1 [17.2, 24.9]	0.675	a
hsCRP (mg/dL)	0.8 [0.3, 3.6]	0.6 [0.3, 1.8]	>0.999	a

Data are presented as mean ± standard deviation or median [interquartile range]

^†^median ΔWC = 2.8%

*p-*value: a, Wilcoxon signed rank test; b, paired t-test

Abbreviations used in this table are the same as in Table 1.

The degree of change in serum total adiponectin levels from baseline to 1 year was significantly larger in the ΔWC < median group (from 7.5 to 8.3 μg/mL) than in the ΔWC ≥ median group (from 8.4 to 8.3 μg/mL) (*p* = 0.017). Moreover, as shown in Table 4, the percent change in the total and HMW adiponectin levels was significantly greater in the ΔWC < median group than in the ΔWC ≥ median group (*p* = 0.011 and *p* = 0.009, respectively). The percent change in HMW/total adiponectin was also significantly greater in the ΔWC < median group than in the ΔWC ≥ median group. After correcting for the number of cigarettes smoked in a day, years of smoking, and sex, the two groups also differed significantly in terms of the percent change in total adiponectin levels from baseline to 1 year after quitting smoking (ΔWC (%) ≥ median group: 10.9 ± 3.5 vs. ΔWC (%) < median group: −1.5 ± 3.5, *p* = 0.016).

**Table 4:** Percent change in patient data from pre- to post- smoking cessation: Comparison between patients with lesser versus greater abdominal obesity.

	ΔWC (%) < median^†^ (n = 39)	ΔWC (%) ≥ median (n = 40)	*p-*value	
**% change BMI**	**1.5 [**−**2.8, 3.7]**	**7.0 [4.0, 11.3]**	**<0.001**	**a**
**% change WC**	−**1.1 ± 3.7**	**8.1 ± 3.7**	**<0.001**	**b**
**% change SBP**	−**4.5 ± 13.3**	**1.9 ± 12.2**	**0.035**	**b**
**% change DBP**	−**3.4 ± 11.8**	**5.3 ± 16.8**	**0.011**	**b**
**% change HbA1c**	−**1.6 [**−**3.9, 1.8]**	**1.7 [0.0, 5.7]**	**0.006**	**a**
**% change LDL-C**	−**6.6 ± 17.5**	**11.3 ± 29.5**	**0.004**	**b**
% change HDL-C	8.8 [−4.5, 21.2]	0.6 [−9.4, 12.8]	0.078	a
**% change TG**	−**2.9 [**−**27.6, 51.1]**	**40.0 [**−**11.9, 94.2]**	**0.012**	**a**
**% change leptin**	**18.1 [**−**13.4, 63.6]**	**90.8 [32.1, 157.6]**	**<0.001**	**a**
**% change total adiponectin**	**10.8 ± 22.9**	−**1.5 ± 19.0**	**0.011**	**b**
**% change HMW adiponectin**	**23.7 [−6.3, 47.7]**	**-2.4 [−17.0, 15.0]**	**0.009**	**a**
**% change HMW/total adiponectin**	**15.3 [−1.8, 27.1]**	**-2.5 [−15.5, 14.8]**	**0.023**	**a**
**%** change hsCRP	0.9 [0.4, 2.7]	0.9 [0.4, 1.8]	0.364	a

Data are presented as mean ± standard deviation or median [interquartile range]

^†^median ΔWC = 2.8%

*p-*value: a, Mann−Whitney U test; b, unpaired t-test

Abbreviations used in this table are the same as in Table 1.

## Discussion

Although the risk for various diseases decreases over a prolonged period after smoking cessation, weight gain is observed for three years after the cessation [20]. Weight gain due to smoking cessation may hamper the reduction in the risk of cardiovascular disease by increasing lipid abnormalities, hypertension, and insulin resistance. Thus, the advantages of smoking cessation and the disadvantages of gaining weight intricately coexist for several years after smoking cessation. α1-antitrypsin-low-density lipoprotein (AT-LDL) is an oxidatively modified LDL that promotes atherosclerosis. We have previously reported that although serum AT-LDL levels improve 3 months after smoking cessation, this improvement is hampered in patients who gain weight after smoking cessation [23]. In addition, we have reported that AT-LDL levels in the blood decrease despite gaining weight over a year after smoking cessation. In other words, the benefits of smoking cessation may surpass the drawbacks of becoming obese thereafter [21]. Nevertheless, precise long-term changes in other cardiovascular indices after smoking cessation and the exact effects of obesity after smoking cessation on indices of cardiovascular risk remain unknown.

Weight typically continues to increase up to three years after an individual quits smoking [20], and our current study confirmed this trend. We also found that weight continued to increase from three months to one year after quitting smoking; conversely, AT-LDL levels decreased (improved) [21]. In contrast, the current study did not find significant differences in adiponectin levels before and one year after quitting smoking. AT-LDL and adiponectin are cardiovascular biomarkers that improve when an individual quits smoking; however, it is thought that adiponectin is affected more by weight gain than by AT-LDL. Cardiovascular biomarkers include markers that are more affected by weight gain and markers that are more affected by quitting smoking. These markers behave differently, involving complex interactions between changes in weight after quitting smoking and temporal factors.

AT-LDL is an oxidized LDL that is primarily related to arteriosclerosis [21]. However, adiponectin is considered to be a “good” cytokine that is secreted by adipose tissues. Adiponectin has beneficial actions, including anti-arteriosclerotic action, action to improve glucose metabolism, antioxidant action, anticancer action, action to inhibit cardiomegaly, and action to provide cardiovascular protection, in a host of lifestyle-related diseases. Adiponectin is a biomarker that is more affected than AT-LDL by changes in weight. We found no significant improvement in adiponectin, indicating that the negative aspects of weight gain after quitting smoking negated the positive aspects of quitting smoking. Nonetheless, our results are the first to show that adiponectin levels may improve if weight gain is inhibited after an individual quits smoking. Our results support the hypothesis that inhibiting and controlling weight gain after quitting smoking is beneficial.

Typically, serum adiponectin levels decrease due to obesity, particularly due to increased visceral fat [16, 24]. Moreover, serum adiponectin levels decrease significantly in conjunction with heavy daily smoking [25]. The mechanism through which adiponectin levels in blood decreases because of smoking is commonly believed to be the one where increased oxidative stress causes decreased adiponectin production [26]. In fact, nicotine promotes the release of TNFα and free fatty acids from adipocytes [27]. In addition, it is reported that exposure to cigarette smoke directly inhibits adipocytes from the extracellular release of multimeric adiponectin [18, 28]. Nevertheless, the precise mechanism by which adiponectin levels in blood decrease because of smoking remains unclear.

A previous study reported that adiponectin level in blood remained elevated for three months after quitting smoking [19]. In addition, another study reported that the adiponectin level in the blood of smokers increased significantly for six months after quitting smoking [29]. Unlike previous studies, significant changes were not noted in adiponectin levels before and one year after quitting smoking. However, correcting for WC at each time of measurement revealed a significant increase in adiponectin levels in the blood after quitting smoking. If a patient’s WC does not change after quitting smoking, it is likely that the adiponectin level will increase significantly because of quitting smoking. Body weight and WC increased from baseline to one year after quitting smoking. Moreover, an examination of the percent increase in WC after an individual quit smoking found no significant improvement in adiponectin levels in participants with increased WC; however, significant improvement in adiponectin levels was noted in participants with no increase in WC. Therefore, an increase in WC may impede an increase in adiponectin levels. If weight gain after quitting smoking is restricted or if an individual continues to refrain from smoking for a prolonged period, adiponectin levels may rise. In fact, a study reported that serum adiponectin levels increase (improve) after smoking cessation but take ≥10 years in women and ≥20 years in men to increase to levels found in nonsmokers [25]. Therefore, long-term changes in adiponectin levels related to weight changes need to be studied further.

Adiponectin levels are affected by factors such as sex and smoking status. After correcting for the number of cigarettes smoked in a day, years of smoking, and sex, the percent change in the blood adiponectin levels from the baseline to one year after quitting smoking was significantly larger in the group with less increase in the WC compared to the group with greater WC increase. Therefore, adiponectin levels in blood increased in participants without abdominal obesity. This finding was unrelated to sex and smoking status.

In the present study, the adiponectin levels did not change 1 year after smoking cessation in individuals with an increased waist circumference. However, total adiponectin levels increased in individuals with a smaller increase in waist circumference after smoking cessation. In other words, smoking cessation promoted the production or secretion of adiponectin in the body, while obesity after smoking cessation inhibited this action. Reports have stated that smoking reduces adiponectin levels in the blood; that is, nicotine, a major component of tobacco smoke, directly inhibit the expression of the adiponectin gene in the adipocytes [18, 30, 31]. When adipocytes are exposed to cigarette smoke, the secretion of adiponectin outside the cells is also inhibited [18, 28]. Adiponectin accumulates in the vascular walls that have been damaged by smoking, and it is then consumed, which results in decreased adiponectin levels in the blood [30, 32]. It is also reported that smoking induces oxidative stress and the production of inflammatory cytokines, such as TNFα. These cytokines inhibits the expression of the adiponectin gene [18, 30, 33]. However, a report has stated that a prolonged period (10–20 years) is required for inflammatory cytokines to decrease after smoking cessation [2]. In fact, our study indicated that the hsCRP level, which is a marker of inflammation, did not change after smoking cessation in both individuals with smaller and larger increases in waist circumference. On the basis of these findings, it is considered that adiponectin in the blood increases 1 year after smoking cessation primarily because smoking cessation releases nicotine-mediated inhibition of adiponectin expression and secretion.

Adiponectin circulates in the blood as a multimer in various molecular forms (high-molecular-weight [HMW] multimers, medium-molecular-weight hexamers, and low-molecular-weight trimers). Among the forms of adiponectin, HMW adiponectin is considered metabolically active [34]. As obesity progresses, medium- and low-molecular-weight adiponectin do not decrease, whereas HMW adiponectin decreases [35, 36]. Low HMW adiponectin level and HMW/total adiponectin are significantly associated with the development of insulin resistance [37] and metabolic syndrome [38]. Smoking decreases adiponectin levels, particularly HMW adiponectin levels, in the blood. HMW adiponectin levels substantially decrease because of heavy smoking [28, 39]. In addition, a report has stated that smoking inhibits the expression of DsbA-L (a protein present in the endoplasmic reticulum that promotes the multimerization and secretion of adiponectin) and the secretion of HMW adiponectin by the cells [28]. The HMW adiponectin levels and the HMW/total adiponectin significantly increased prior to smoking cessation until 1 year afterward in individuals with a smaller increase in waist circumference, but not those with a larger increase of waist circumference in this study. The detailed mechanism associated with this increase is unclear. However, our results revealed that HMW adiponectin is the primary driver for the increase in total adiponectin levels. HMW adiponectin levels are reported to be inversely correlate with cardiovascular risk [34]. Therefore, our results suggest that smoking cessation reduces cardiovascular risk and that obesity after smoking cessation inhibits the reduction in cardiovascular risk.

The current study found that compared to those with increased WC, individuals with no increase in WC 1 year after smoking cessation had lower blood pressure, higher HDL-C (the “good” cholesterol) levels, and significantly higher serum adiponectin levels despite having a BMI that was the same (or a slightly increased) as that prior to smoking cessation. Cardiovascular risk takes ≥10 years to improve to levels found in nonsmokers [1]. However, if abdominal obesity is curtailed, then the current findings suggested that a reduction in cardiovascular risk may be apparent 1 year after smoking cessation. The current results revealed no changes in high-sensitivity C-reactive protein (hsCRP) levels one year after quitting smoking. A study reported that CRP levels gradually decrease five years after quitting smoking [40]. Another study reported that after an individual quits smoking, hsCRP levels may take 20 years to reach the same levels found in nonsmokers [2]; therefore, inflammatory markers improve slowly over a prolonged time period. In contrast to inflammatory markers, AT-LDL levels improved in just three months. The current results revealed no decrease improvement in adiponectin levels one year after quitting smoking. Evidently, levels of multiple markers related to cardiovascular risk change at different times after quitting smoking.

Normally, serum adiponectin levels decrease due to obesity. However, such a decrease had not been observed in individuals with increased WC 1 year after smoking cessation. Thus, the advantages of smoking cessation may cancel out the disadvantages of an increase in WC in patients who become obese 1 year after smoking cessation.

Studies have noted an average weight gain of about 4–5 kg after smoking cessation, while others have reported that weight is readily gained by heavy daily smokers [41] or individuals with a high level of nicotine dependence (FTND score) [42]. Obesity has a substantial effect on the prognosis of various diseases. Hence, particular attention must be paid to weight gain after smoking cessation [43]. Nevertheless, weight gained after smoking cessation is primarily due to subcutaneous fat and not visceral fat [44, 45]. In addition, weight gained due to smoking cessation includes an increase in not only fat but also bone and muscle tissues [46]. The nature of the weight gained is probably one reason why a decrease in adiponectin levels was not noted in individuals with increased WC.

Leptin inhibits appetite by acting on the satiety center in the hypothalamus, which activates the sympathetic nervous system causing the body to burn fat and control obesity [24]. Studies have noted that patients who are obese typically have decreased leptin receptor signaling and leptin resistance, resulting in increased leptin levels in the blood [47]. Consistent with previous studies, the current study noted a significant increase in serum leptin levels after smoking cessation [48–50]. After considering changes in WC, we found no significant changes in leptin levels in individuals with no increase in WC after smoking cessation but a significant increase in leptin levels in individuals with abdominal obesity. Thus, abdominal obesity presumably caused leptin resistance despite smoking cessation. The effects that smoking cessation has on leptin are still unclear, although previous studies have indicated that leptin levels are independent of smoking status [50]. At least in individuals with abdominal obesity after smoking cessation, increased leptin levels are probably affected more by weight gain after smoking cessation than by changes in smoking status.

A previous study reported that insulin resistance improved after quitting smoking. If weight gain after quitting smoking is minimized, insulin resistance is likely to improve. In fact, the present study results revealed no significant increase in blood levels of leptin, which reflects instant resistant, in participants with no increase in WC after quitting smoking. In future, long-term changes in insulin resistance need to be studied to better ascertain how insulin resistance changes after an individual quits smoking.

### Limitations

The current study noted obvious improvements in markers of cardiovascular risk, such as blood pressure, HDL-C levels, and adiponectin levels, in individuals with no increase in WC. This finding suggests that preventing abdominal obesity after smoking cessation may limit cardiovascular disease risk. Nevertheless, whether preventing obesity after smoking cessation via an intervention, such as nutritional guidance, would actually lead to a lower risk for developing cardiovascular disease needs to be studied further in the future.

## Conclusions

Despite weight gain and increased abdominal obesity, serum adiponectin levels did not decrease after smoking cessation. Thus, the beneficial effect of smoking cessation may eliminate the adverse effects of weight gain thereafter. On the other hand, serum adiponectin levels increased in patients with less abdominal obesity 1 year after smoking cessation. Therefore, in such patients, the beneficial effect of smoking cessation on adiponectin levels becomes apparent.
